# Antioxidant and anti-inflammatory activity of a nanoparticle based intracanal drugs

**DOI:** 10.6026/97320630018450

**Published:** 2022-05-31

**Authors:** Iffat Nasim, Marimuthu Shamly, KrishnaKanth Jaju, Veeraraghavan Vishnupriya, Zohra Jabin

**Affiliations:** 1 Department of Conservative Dentistry and Endodontics, Saveetha Dental College and Hospitals, Saveetha Institute of Medical and Technical Sciences, Saveetha University, India; 2 Department of Biochemistry, Saveetha Dental College and Hospitals, Saveetha Institute of Medical and Technical Sciences, Saveetha University, Chennai - 600 077, India; 3 Divya Jyoti College of Dental Sciences, Modinagar, Uttar Pradesh, India;

**Keywords:** Antioxidant, anti-inflammatory, activity, nanoparticle, intracanal drugs

## Abstract

The most common intracanal medication is calcium hydroxide. Its efficacy can be affected by a number of factors, including pH, serum proteins, collagen, and dentin. It's also ineffective against E. faecalis and fungus, lacks an anti-inflammatory component, and has mixed reviews when it comes to pain relief. Natural alternatives to synthetic intracanal medication are being researched at the moment. We evaluated the antioxidant and anti-inflammatory activity of green synthesized silver nanoparticle based intracanal medicaments. Silver nanoparticles integrated into calcium hydroxide and graphene oxide nanoparticles were the experimental groups and Calcium hydroxide served as the control. Antioxidant activity was determined using the DPPH and Nitric oxide assays, while anti-inflammatory activity was determined using the protein denaturation and Xanthine Oxidase Inhibition assays. Both experimental groups had higher antioxidant activity than the control group based on DPPH and Nitric oxide assays. Calcium hydroxide combined with silver nanoparticles demonstrated improved anti-inflammatory efficacy in a protein denaturation and Xanthine oxidase inhibition assay. Within the constraints of an in vitro study, it can be concluded that intracanal medicaments containing silver nanoparticles can be employed efficiently during root canal preparation. In comparison to standard calcium hydroxide-based intracanal medicaments, it has effective antioxidant and anti-inflammatory effects.

## Background:

Intracanal medications are a key aspect of root canal therapy and are considered a fundamental part of the treatment. Inter-appointment antimicrobial medication works by preventing bacteria from multiplying and eliminating those that survive, as well as reducing pathogen infiltration as a result of micro leakage. The purpose of an intracanal dressing is to prevent microbial repopulation between the visits in multiple appointment root canal therapy [1]. In the treatment of cases with pulpal necrosis and apical periodontitis, the role of intracanal medicaments becomes more important and challenging. The research shows that even after chemo-mechanical preparation, most root canals retain live bacteria [2]. Because of the greater total time spent on the therapy, it is probable that utilizing intracanal medicaments will result in a more complete instrumentation [3]. Nanoparticles improve solubility, bonding, chemical activity, and antibacterial efficiency of intracanal medicaments by increasing their surface-to-volume ratio [4]. When utilized as an intracanal medicament, silver nanoparticles have been shown to be effective against bacteria and biofilms [5]. Antimicrobial activity of biosynthesized AgNPs against E. faecalis biofilm on root dentin is evident [6]. Graphene, an organic nanoparticle, is the thinnest substance and an allotrope of carbon. It has outstanding antibacterial properties [7]. Antioxidants may improve the binding of obturating materials to root canal dentin by allowing sealers to penetrate deeper into the dentinal tubules due to increased polymerisation [8]. It can repair root dentin damage caused by instrumentation and irrigation [9]. Most root canal patients have pain between sessions or after the treatment as a result of the underlying inflammation, which can be avoided by utilising anti-inflammatory medications [10]. Therefore, it is of interest to determine the antioxidant and anti-inflammatory property of green synthesized silver nanoparticle incorporated intracanal medicament.

## Material and Methods:

Fresh leaves of Andrographis paniculata and Ocimum sanctum Linn were collected and dried for three days. After that, they were coarsely pulverized. 1g powdered Andrographis paniculata and 1g powdered Ocimum sanctum Linn leaves were weighed and dissolved in 200ml distilled water before being thoroughly blended. Using a heating mantle, it was then boiled for 5 minutes at 60-80°C. The supernatant of the boiling extract was filtered through Whatman No.1 filter paper and utilized.

## Preparation of silver nanoparticles:

>20 ml of pure plant extract was blended thoroughly into 180 ml of 1 mM silver nitrate solution. The solution was then kept in an orbital shaker to mix further. The color of the prepared solution gradually changed to dark brown as the visual changes were seen. This showed the synthesis of silver nanoparticles. To validate the synthesis of nanoparticles, the produced solution was analyzed using a UV Vis Spectrophotometer. The solution was then centrifuged for 10 minutes at 8000 rpm. After that, the solution was filtered through Whatman No. 1 filter paper.

## Preparation of Graphene oxide nanoparticles:

In 100 mL of distilled water, 1.2g of graphite nano powder (Sisco Research Laboratories, Maharashtra, India) and 0.4 g of sodium hydroxide (MERCK, Mumbai, India.) were dissolved; to this, 100 mL of plant extract was added and thoroughly mixed. The solution was then maintained for additional mixing in an orbital shaker and magnetic stirrer with a heated plate. The color shift was recorded, and the development of nanoparticles was observed. The solution was then centrifuged for 10 minutes at 8000 rpm. After that, the solution was filtered through Whatman No. 1 filter paper. Successful synthesis of nanoparticles was done using UV Visible Spectrophotometer analysis, TEM Analysis, X-ray Diffraction and FTIR analysis. Antioxidant and anti inflammatory activity tests were performed on the nanoparticles that had been synthesized.

## Assessment of antioxidant activity:

All chemicals and reagents used in this investigation were acquired from Sigma Chemicals Company, St. Louis, MO, USA, and Sisco Research Laboratories (SRL), Mumbai, India.

## DPPH free radical scavenging activity:

A 1.0 ml DPPH solution was added to 1.0 ml of each medicament at various concentrations (0.1, 0.2, 0.3, 0.4, and 0.5 mg/ml). The activity was measured at 517 nm after the combination was held at room temperature for 50 minutes. As a standard, the same amounts of ascorbic acid were utilized. The ability to scavenge the DPPH radical was determined and expressed as a percentage of inhibition.

## Nitric oxide radical scavenging activity:

2ml sodium nitro-prusside, 500l phosphate buffered saline (PBS), and 500l of different concentrations (0.1, 0.2, 0.3, 0.4, and 0.5 mg/ml) of test drug were mixed in a total volume of 3ml reaction mixture and incubated for 1 hour 30 minutes at 25°C. Then, for complete diazotization, 500l of nitrite-containing reaction mixture was combined with 1 ml of sulfanilic acid and allowed to stand for 5 minutes. Then 1 mL of naphthyl ethylene diamine dihydrochloride was added, stirred, and set aside at 25°C for 30 minutes. In scattered light, a pink tinted chromophore forms. As a standard, the same amounts of ascorbic acid were utilized. At 550 nm, the activity was measured and the results were represented as a percentage of inhibition.

## Assessment of Anti inflammatory activity:

### Protein denaturation assay:

In Tris Buffered Saline, a BSA solution (0.4 percent, w/v) was produced (one tablet is dissolved in 15 mL of deionized water to yield 0.05M Tris and 0.15M sodium chloride, pH 7.6 at 25°C). Glacial acetic acid was used to alter the pH to 6.4. Each medicament with concentrations of 0.1mg/ml to 0.5mg/ml was added to test tubes containing 1 mL of 0.4 percent, w/v BSA buffer solution. In the same way, both negative (methanol) and positive (aspirin) controls were tested. Under laboratory conditions, the solutions were heated for 10 minutes in a water bath at 72°C and then cooled for 20 minutes. Using an air blank, the turbidity (level of protein precipitation) of the solutions was measured at 660 nm in a Hach Spectrophotometer. The studies were repeated twice, with the average absorbance values reported. On a percentage basis, the percentage blockage of precipitation (protein denaturation) was calculated.

*% Anti-Denaturation Activity = Absorbance of control - Absorbance of sample x 100 Absorbance of control.

### Xanthine oxidase inhibitory activity:

Xanthine oxidase enzyme (Sigma Aldrich) was mixed with bovine milk, and then the enzyme was diluted in a solution to a concentration of 2 units/ml. To improve the solubility of the xanthine substrate solution, 5 drops of 1.0 M NaOH were added, followed by the production of a 1 mM xanthine solution. The drugs were dissolved in 1 percent dimethyl sulfoxide (DMSO) and diluted to achieve final concentrations of 0.1mg/ml to 0.5mg/ml. As a positive control, allopurinol was employed as a reference medication. The assay combination had a total volume of 3.2 mL and included 1 mlof tested drug, 1 mL 0.15 M phosphate buffer (pH 7.8), and 100 mL enzyme xanthine oxidase solution. After a 15-minute pre-incubation period at 37°C, the reaction was started by adding 100 L of xanthine substrate solution and incubating at 37°C for 30 minutes. 1 mL of 1N HCl was used to stop the process. The production of uric acid was determined by measuring the absorbance at 295 nm. The absorbance of uric acid from the assay combination without test extract (blank sample) and with test extract was measured to estimate the percent of xanthine oxidase inhibitory activity of the tested samples. The IC50 values were calculated using a linear regression analysis of several different sample concentrations vs percent inhibition.

## Results:

### DPPH assay:

Percentage of inhibition of all the drugs increased with increase in concentration and was comparable with the control ascorbic acid. For AgCaOH the maximum percentage of inhibition was 82%, Ag GO- 92.5%, CaOH-80.5% (Table 1 -see PDF).

### Nitric oxide assay:

Percentage of inhibition of all the drugs increased with increase in concentration. For AgCaOH the maximum percentage of inhibition was 84%, Ag GO-73%, CaOH-67% (Table 2 - see PDF).

### Protein denaturation assay:

Protein denaturation activity of all the drugs increased with increase in concentration. For AgCaOH the maximum denaturation was observed to be 84%, AgGO-76.5%, CaOH-78.5% (Table 3 - see PDF).

### Xanthine oxidase assays:

Percentage of inhibition of all the drugs increased with increase in concentration. For AgCaOH the maximum percentage of inhibition was 80%, Ag GO-68.5%, CaOH-70.5% (Table 4 - see PDF).

## Discussion:

Calcium hydroxide has been considered as the gold standard intracanal medicament utilised today; yet, as endodontics has progressed, newer materials have emerged [11]. The persistence of microbes might be regarded as the primary reason for root canal failure [12]. The capacity of E. faecalis to enter into the dentinal tubules and resist bactericidal chemicals has been attributed for the organism's involvement in recurrent root canal infections [13]. This has prompted extensive research in the field of endodontics to find an alternate intracanal medicament. Because of their good electrical conductivity, chemical stability, and antibacterial activity, silver nanoparticles have gained a lot of attention [14]. When utilized as an intracanal medicament, silver nanoparticles have been shown to be effective against bacteria and biofilms. Antimicrobial activity of biosynthesized AgNPs against E. faecalis biofilm on root dentin has been demonstrated [15]. Graphene is a two-dimensional hexagonal carbon-based flat monolayer with unique mechanical, electrochemical, and physical properties. This organic nanoparticle is an allotrope of carbon and is the thinnest material, It has excellent antimicrobial properties [16] [17]. Using leaf extracts from Andrographis paniculata and Ocimum sanctum Linn, silver and graphene oxide nanoparticles were produced. The anti-inflammatory properties of Andrographis paniculata are well established [18]. Upper respiratory infections, intestinal infections, and renal calculi are all treated with Ocimum sanctum Linn [19]. One of the most effective methods for disinfecting the canals and controlling pain is to place biocompatible drugs that have either a potent antibacterial component or an anti-inflammatory component, or both, into the root canals [20]. In this present study both the experimental groups had higher antioxidant activity than the control group based on DPPH and Nitric oxide assays. Calcium hydroxide combined with silver nanoparticles demonstrated improved anti-inflammatory efficacy in protein denaturation and Xanthine oxidase inhibition testing. Cell walls of pathogens are oxidized and denatured by silver nanoparticles, leading to cell lysis [21]. Graphene causes oxidative stress by causing mechanical stress, extracting phospholipids from lipid membranes, and generating reactive oxygen species [22]. As a reducing agent, plant extracts from Andrographis paniculata and Ocimum sanctum Linn were utilized. The high phenolics and flavonoids content in the plant may be responsible for the antioxidant efficacy of AgNP in this investigation [23]. Because they are electron donors and play a major role in scavenging and neutralizing free radicals, these plant phenolics have a high reduction capacity and are potent antioxidants [24]. The generation of silver and graphene oxide nanoparticles with extra capabilities provided by the capping of phytochemicals can be done in an environmentally safe and cost-effective way using phytochemicals. Alkaloids cover the silver nanoparticles, preventing them from aggregating and stabilizing them promoting anti inflammatory activity [25]. Finally, combining the advantages of phytomedicine with nanomedicine can lead to the development of more efficient silver and graphene oxide nanoparticles with less harmful consequences.

## Conclusion:

The leaf extracts of Andrographis paniculata and Ocimum sanctum Linn were utilized for green synthesis of silver and graphene oxide nanoparticles in the present study. Within the constraints of this investigation, it can be concluded that intracanal medicaments based on silver nanoparticles can be employed efficiently during root canal preparation. In comparison to standard calcium hydroxide-based intracanal medicaments, it has effective antioxidant and anti-inflammatory effects. More in vivo research is needed to support the findings.
